# Prognostic Value of Histological and Immunohistochemical Data in Diabetic Foot Ulcers

**DOI:** 10.3390/jcm11237202

**Published:** 2022-12-04

**Authors:** Konstantin Koreyba, Ekaterina Silina, Dmitry Tsyplakov, Petr Litvitskiy, Natalia Manturova, Zalim Balkizov, Raghu Ram Achar, Nithya Rani Raju, Victor Stupin

**Affiliations:** 1 Department of Surgical Diseases, Kazan State Medical University, 420012 Kazan, Russia; 2 Institute of Biodesign and Modeling of Complex Systems, I.M. Sechenov First Moscow State Medical University (Sechenov University), 119991 Moscow, Russia; 3 Department of General Pathology, Kazan State Medical University, 420012 Kazan, Russia; 4 Department of Plastic and Reconstructive Surgery, Cosmetology and Cell Technologies, Pirogov Russian National Research Medical University, 117997 Moscow, Russia; 5 Department of Hospital Surgery, Pirogov Russian National Research Medical University, 117997 Moscow, Russia; 6 Division of Biochemistry, School of Life Sciences, JSS Academy of Higher Education & Research, Mysuru 570015, Karnataka, India

**Keywords:** diabetic foot ulcer, diabetic foot syndrome, amputation, primary surgical treatment, wound healing, immunohistochemistry, prognosis, diabetes, fibroblasts, neutrophilic leukocytes, endothelocytes

## Abstract

Diabetic foot ulcers are an extremely urgent medical and social problem throughout the world. The purpose of this study was to analyse the histological and immunohistochemical features of tissues and cells of different sections of wounds taken during the primary surgical treatment of chronic wounds in patients with diabetic foot syndrome with favourable and unfavourable outcomes. Material and methods. A clinical prospective observational study of the treatment outcomes of fifty-three patients with diabetic foot ulcers hospitalized twice in one specialized centre over the course of the year was conducted. The analysis of histological and immunohistochemical data of the tissues of the edges and the centre of the ulcer taken during the primary surgical treatment was performed. While performing histological analyses of wound tissues, special attention was given to the determination of cellular characteristics of leukocyte-necrotic masses, granulation tissue, and loose and dense connective tissue. Immunohistochemistry was performed using a set of monoclonal antibodies, allowing verification of neutrophilic leukocytes, fibroblasts, and endothelial cells. Results. Unfavourable outcomes (amputation, reamputation, death from cardiovascular diseases, nonhealing ulcer within a year) were registered in 52.8% of cases. Uniform distribution of neutrophils and endothelial cell fibroblasts in all parts of the wound was recorded in patients with a favourable outcome. An unfavourable outcome was predetermined by the uneven content of these cells with a significant increase in neutrophilic leukocytosis in the bottom of the wounds, as well as a significant decrease in the number of fibroblasts and endotheliocytes in the centre of the wounds. Conclusions: The datasets obtained during primary surgical treatment are extremely informative to predict the outcome of the treatment of diabetic foot ulcers and indicate more active surgical strategies with the potential to reduce the treatment time, increase its effectiveness, and eventually make the treatment cost-effective.

## 1. Introduction

Diabetes is an extremely urgent medical and social problem. Despite the understanding of the general mechanisms of its course and the developed rules of treatment accepted by the medical community, type 2 diabetes remains one problem that cannot be completely dealt with despite the constant search for new drugs and approaches to prevention and treatment [1,2,3,4]. The number of such patients is rapidly increasing, as is the number of complications associated with the disease [5,6,7,8,9]. One of these complications, which permanently disables a person and is accompanied by the appearance of nonhealing or poorly healing ulcers on the distal parts of the lower extremities, is known in the medical literature as diabetic foot ulcers or diabetic foot syndrome (DFS) [10,11,12,13], which is the leading cause of non-traumatic lower extremity amputation [14,15,16,17,18].

Important not only from a medical but also from an economic point of view is the issue of decision-making for the personalized management of patients with DFS based on a reasoned treatment prognosis [19,20,21,22,23]. The clinical and laboratory studies accepted today—the need for which no one doubts—unfortunately, cannot accurately predict the outcome of the treatment of DFS. The prognostic models of amputation due to DFS developed by different authors contain different prognostic predictors [15,16,17,18]. Thus, there is a need to search for other additional indicators that we can obtain during the medical management of patients with this pathology. One of these methods, perhaps, is the histomorphological study of the edges of a chronic ulcer obtained during the primary surgical treatment of a wound, which is standard in the management of these patients. This biological material can provide rich information and a more accurate personal prognosis, even for the long term.

The purpose of this study was to analyse the histological and immunohistochemical features of tissues and cells of different sections of wounds taken during the primary surgical treatment of chronic wounds in patients with diabetic foot syndrome with favourable and unfavourable outcomes.

## 2. Materials and Methods

A clinical, prospective, observational analytical study of the results of the treatment of patients with diabetic foot syndrome was performed. The material was collected in one specialized centre for diabetic foot in Kazan City, which is a member of the clinics of Kazan State Medical University (the head of the Centre “Diabetic Foot” is Konstantin Koreyba).

This study is part of a large multidisciplinary study on the treatment of type II diabetes mellitus, including diabetic foot, based out of the Kazan State Medical University and the Russian National Research Medical University, conducted from 2014 to 2020. The topic of this study, “Modern high-tech approaches to the diagnosis and treatment of patients with diabetic foot syndrome”, was approved and approved by the Local Ethics Committee of the State Budgetary Educational Institution of Higher Professional Education “Kazan State Medical University” of the Ministry of Health of the Russian Federation (Protocol No. 8 dated 28 October 2014).

The inclusion criteria for this study were a long-term nonhealing ulcer of DFS (more than 6 months) with a Wagner grade II–IV lesion at the level of the toes and foot. The duration of diagnosed diabetes mellitus with a constant intake of oral anti-diabetic agents and/or insulin was at least 6 months.

The patients participating in this study were followed up for a year, including remotely using public internet channels, with each patient being hospitalized at least twice (the second time on average 3 months after the first hospitalization). Then, 12–14 months after the first hospitalization, a catamnesis was obtained (telephone interview with the patient and/or his/her relatives).

Taking into account that patients were planned for a rather long dynamic follow-up, the study included only patients who were adherent to treatment and who had every opportunity to provide themselves with a sufficient amount of modern dressings (ready-to-use hydroactive dressings based on superabsorbents and protected hydrophilic sponge dressings) and patients who can communicate online with their doctor during follow-up. Asocial people and those who did not meet the specified requirements were not included in the study.

The analysis included only patients without critical narrowing of the main arteries of the lower extremities, which was assessed by ultrasonic dopplerography (USDG) of the leg vessels. Thus, patients with critical ischaemia who needed to undergo vascular operations on the main arteries for restoring blood flow in the vessels of the lower extremities were excluded from the study. In the analysed group, there were no cases of massive or deep damage to the bones of the foot. Therefore, surgical treatment of osteomyelitis of the middle and proximal parts of the foot was aimed at the maximum possible preservation of the architectonics of the foot, which was achieved by performing economical osteonecrectomy in combination with prolonged systemic antibiotic therapy, and in the case of dry toe gangrene, disarticulation.

The duration of this study was from April 2018 to March 2020. The COVID-19 pandemic did not affect the results of the study, since the study was terminated on 28 March 2020, that is, before the start of the pandemic and the first lockdown in Russia.

Our study is devoted to the analysis of histological and immunohistochemical parameters of the material obtained during the primary surgical treatment.

### 2.1. Primary Surgical Treatment Technique

**Figure 1:**
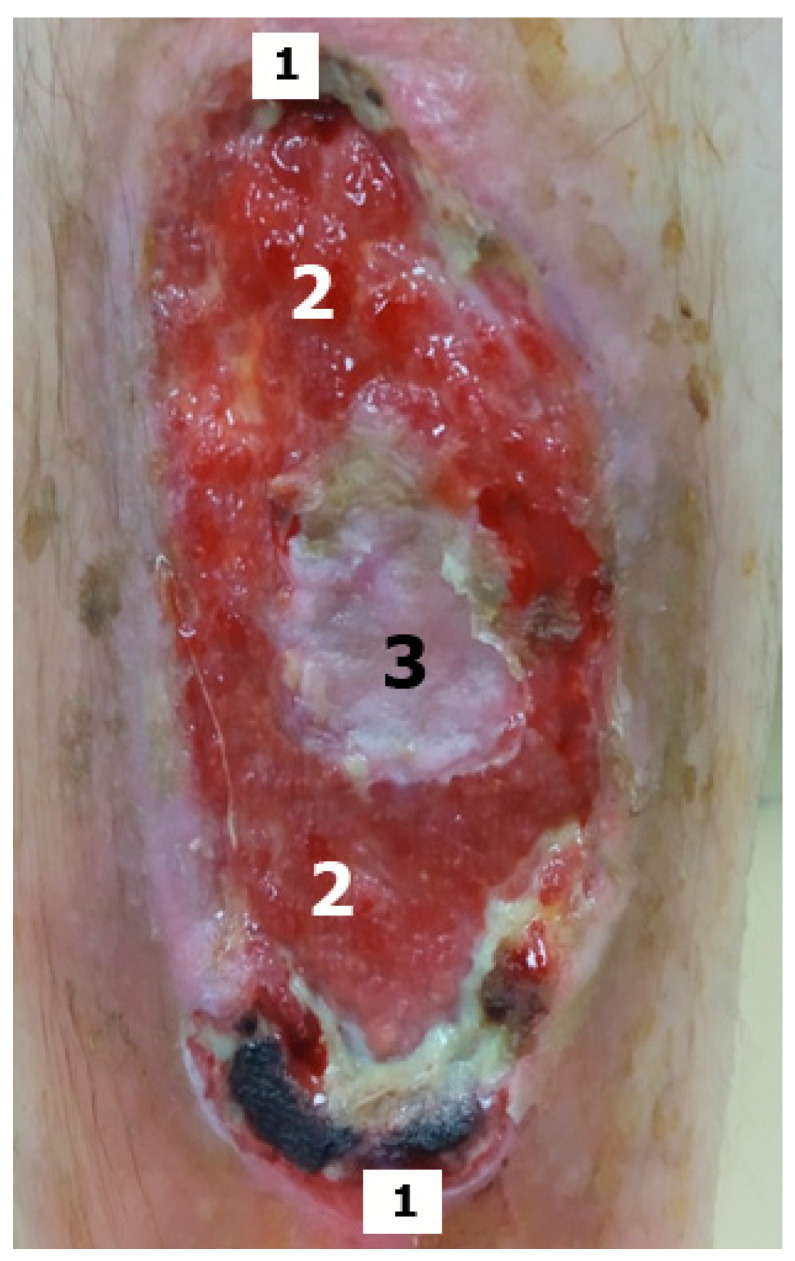
Diabetic foot ulcer and biomaterial sampling sites for histological examination. (1—the edges of the wound defect, 2—the walls of the defect, 3—the bottom of the wound defect). An image of DFS shows that when tissue is taken for histological examination only, for example, from one lower edge, then necrosis and fibrin (inflammation) will be visualized in the morphological material, and proliferative-regenerative processes will be visualized in the opposite sections. The photo also clearly shows the process of epithelialization in the centre of the wound.

The material was taken after three sessions of treatment of the surgical field with an alcohol solution of chlorhexidine and exposure under conduction anaesthesia of the surgical field area with a solution of 2% lidocaine +0.5% Novocaine. The biomaterial was taken with a scalpel with a No 15 or No 15C blade with excision from the area of the edges of the defect, walls, and bottom (centre) of the wound. At the edges of the wounds, sampling was carried out simultaneously from opposite structures (180° angle) with subsequent averaging of the morphometry results. We obtained biopsy samples from three sections of the wound defect due to the heterogeneity of tissue defects in chronic diabetic ulcers (Figure 1).

### 2.2. Methods for Performing Histological and Immunohistochemical Studies

The biopsy sample was fixed in 10% Lilly’s neutral formalin or Bowen’s fluid and, after appropriate wiring, embedded in paraffin. Histological sections were stained with haematoxylin and eosin.

For the immunohistochemical studies, paraffin sections were spread in a Histobalt LEICA HI 1210 water bath, placed on glass slides treated with poly-L-lysine, and dried at 35 °C for one hour. Dewaxing in o-xylene (2 min) was followed by washing and dehydration in two 96% alcohol solutions for 5 min each, followed by 70% alcohol (10 min). The slides were then washed in distilled water, followed by antigen unmasking in citrate buffer (DAKO: Target Retrieval Solution, pH 6.0, code S 169984-2) in a water bath at 95 °C (40 min). After cooling the preparations in the same solution to room temperature and treatment in Tris buffer (TBS, pH 7.4), endogenous peroxidase was suppressed with a 3% hydrogen peroxide solution (20 min). This was followed by a second wash in Tris buffer, after which the sections were left in the refrigerator at a temperature of 4 °C for 12 h.

Immunohistochemistry was performed using a set of monoclonal antibodies (MABs). For the verification of neutrophilic leukocytes, myeloperoxidase, clone RB-373-A, at a working dilution of 1:800 (Dako, Santa Clara, CA, USA), was used as an antigen. Vimentin antigen, clone V9, at a working dilution of 1:300 (Lab Vision, Värmdö, Sweden) was used to identify fibroblasts. Endotheliocytes were verified using CD 31, clone 9611, at a working dilution of 1:20 (BioGenex, Fremont, CA, USA).

The first antibodies were diluted with a special buffer with a component preventing the nonspecific binding of antibodies (DAKO; Antibody Diluent with Background Reducing Component, code S3002). The exposure of the first MABs was 1 h at a constant (30 °C) temperature maintained by a heating plate, LEICA HI 1220 histoplate (Leica Biosystem, IL, USA). Then, the slides with sections were washed for 10 min in Tris buffer. The binding of the first antibodies to cellular and structural elements was determined using the standard biotin-streptavidin-peroxidase method, DAKO: LSAB^®^ + System-HRP, code K0690 (Dako, Santa Clara, CA, USA) with diaminobenzidine as a chromogen. After washing in distilled water, the preparations were additionally stained with Mayer’s haematoxylin for 1–2 min. This was followed by repeated washing in water (15 min), dehydration in 96% alcohol (10 min), and clarification in carbol-xylene (5 min). Sections were mounted in Canadian balsam or special media from DAKO, Ultramount, Faramount, code S302580-2 (Dako, Santa Clara, CA, USA).

The nature of immunohistochemical reactions was assessed, taking into account the intensity and percentage of stained cells.

On histological sections, the areas of the structural components of the tissue (leukocyte-necrotic masses, granulation tissue, connective tissue, individual groups of epithelium, as well as the formed stratified squamous keratinizing epithelium) and the content of certain cellular elements (immunohistochemically labelled fibroblasts, neutrophilic leukocytes, endothelial cells) were determined. To determine the area of the structures, a morphometric grid of random step size created by was used, and for counting cells, a morphometric ocular grid created by G.G. Avtandilov was used [24,25].

The principle of working with a morphometric grid of random step size is as follows: the grid is applied directly to the micropreparation, and under low magnification (×7 eyepiece, ×10 lens), the number of grid intersections per studied structure is counted. The position of the grid on the histological section is randomly changed several times, and each time, the count was repeated. The total number of analysed fields is at least 1000. The total number of intersections of the grid obtained as a result of the calculation pertaining to the entire slice is taken as 100%. Then, the number of grid intersections corresponding to each of the studied structures is converted into percentages.

**Figure 2:**
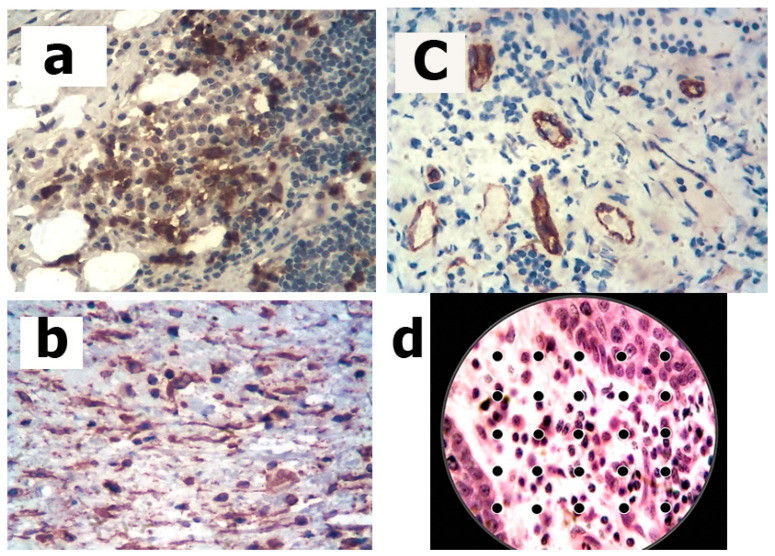
Examples of immunohistochemical identification of neutrophils, fibroblasts, and endotheliocytes, as well as a 25-point morphometric grid for counting cells in the preparation. (**a**) Accumulation of neutrophilic leukocytes in the cell infiltrate. Reaction with MABs against myeloperoxidase. LSAB method with additional staining with hematoxylin: ×400. (**b**) Fibroblasts in cellular infiltrate. Reaction with MABs against vimentin. LSAB method with additional staining with hematoxylin: ×400. (**c**) Clusters of endotheliocytes and small vessels of the capillary type. Reaction with MAb against CD 31. LSAB method with additional staining with hematoxylin: ×400. (**d**) Morphometric reticle of G.G. Avtandilov when counting cellular composition. Stained with hematoxylin and eosin: ×800.

For a quantitative study of the cellular composition, we used the morphometric grid. The working principle of this grid is as follows. First, the grid is fixed inside the eyepiece of the microscope. It contains 25 or 100 points that are visualized in the field of view when viewing a micropreparation. The objects are studied under high magnification: eyepiece ×10, objective ×40 or ×90 (immersion). The number of points per individual cellular element is counted. The field of view of the micropreparation changes (movement of the slide), each time with repetition of the count. Thus, 1000 points per different cell are taken into account. The total number of points (1000) is taken as 100%. The percentage of different cell types is then calculated (Figure 2).

### 2.3. Outcome (Primary Endpoint)

The primary endpoint of the study was a favourable or unfavourable outcome (binary parameter). An unfavourable outcome was one or more of the following events: amputation of a limb due to DFS, death of the patient due to circulatory disease (cardiovascular events, heart failure, myocardial infarction, cerebral stroke) with an unhealed ulcer, and nonhealing of wounds or worsening of the wound process despite the attempted treatment methods. Cases not related to the above accompanied by healing (complete epithelialization) or a significant reduction in the area of the wounds were considered a favourable outcome. The outcome was ranked in the database as a binary variable: 0—favourable, 1—unfavourable outcome.

### 2.4. Statistics

Statistical data analysis was conducted using Statistical Package for the Social Sciences SPSS 25.0 software (IBM Company, New York, NY, USA).

Descriptive statistics of continuous variables are presented as the median (Me) and interquartile range (IQR) if they presented a non-normal distribution. The interquartile range in the tables includes Tukey’s folds. The normality of the distribution of continuous quantitative variables was evaluated using the Kolmogorov–Smirnov test. Descriptive statistics for categorical variables are presented as absolute counts with percentages, *n* (%).

To compare two independent continuous indicators of samples with nonnormal distributions, the Mann–Whitney U test was used. To compare dependent nonparametric samples, the Friedman test was used (histological composition of cells from 3 wound sections). For multiple pairwise comparisons, the Bonferroni correction was introduced into the significance calculations. To check for differences in the proportions of qualitative categorical variables, the chi-squared test, analysis of contingency tables, or Fisher’s exact test was used.

Correlation analysis of quantitative continuous and ordinal variables was carried out according to the Spearman method.

For all tests, statistical significance was set at *p* = 0.05.

## 3. Results

### 3.1. Clinical Characteristics of Patients with Diabetic Foot Ulcers Included in the Study

The study included 53 patients (20 (37.7%) men and 33 (62.3%) women) aged 30 to 82 years (mean age 63 years, IQR 57–67 years) with type II diabetes mellitus and diabetic foot ulcers. The distribution of patients by pathogenetic type of DFS revealed that 32% had neuropathic DFS, 43.4% had ischaemic DFS, and 24.5% had mixed forms of DFS. The duration of diagnosed diabetes mellitus ranged from 0.5 to 34 years up to the time of the first hospitalization and inclusion of patients in the study.

Only 14 (26.4%) patients had higher education. The rest had secondary or specialized secondary education (73.6%). This is consistent with the opinion of other researchers in assessing the importance of education in the meaningful implementation of a healthy lifestyle [26,27,28,29]. Most of the patients were overweight, which is also in line with global trends [30,31,32]. Body mass index (BMI) was below 25 kg/m^2^ in only 9 (17.0%) patients, was in the range of 25–30 kg/m^2^ for 38 (71.7%) patients, and 6 patients (11.3%) were obese and had a BMI above 30 kg/m^2^.

All patients had previously been hospitalized for DFS with a depth of Wagner grade II–V lesion at the level of the toes (*n* = 5, 9.4%) and foot (*n* = 48, 90.6%).

When first included in the study, 17% of patients moved independently, 56.6% used a support (cane, crutches, etc.) for movement, and 26.4% were hospitalized in a wheelchair. The most common general complaints of the patients were numbness (83%) and leg cramps (77.4%). On examination, for 94.3% (*n* = 50) of patients, the foot was oedematous, and for 46 (86.8%), the oedema was pronounced. In 44 (83.0%) patients, the leg affected with DFS was accompanied by hyperaemia. The colour of the feet was normal in only 5 (9.4%) patients; in 27 (50.9%) patients, the feet were pale; and in 21 (39.6%) patients, the feet were bluish in colour. Hyperkeratosis of the affected lower extremity was registered in 37 (69.8%) patients, and deformity of the toes and/or foot was registered in 35 (66.0%) cases.

Among the comorbidities in our patients, diseases of the circulatory system were the most common. Forty-eight (90.6%) patients suffered from arterial hypertension, ten (18.9%) patients had a history of myocardial infarction, and one (1.9%) had a cerebral stroke. Ischaemic heart disease without postinfarction cardiosclerosis was diagnosed in 33 (62.3%) patients, chronic heart failure in 37 (69.8%), and chronic cerebral ischaemia of vascular origin in 27 (50.9%).

Diabetic retinopathy was diagnosed in 37 (69.8%) patients, nephropathy in 28 (52.8%), and diabetic neuropathy in 49 (92.5%).

### 3.2. Assessment of the Frequency of the Primary Endpoint (Adverse and Favourable Outcomes)

Limbs were amputated in 17 (32.1%) patients (in 5 people, only during the first hospitalization; in 5 people, only during the second hospitalization; and in 7 patients, amputation was performed both during the first and second hospitalizations with an increase in the level of amputation of the leg). As a result, the height of the amputations per year was at the level of the toes in seven (13.2%) patients, at the level of the foot in seven (13.2%) patients, at the level of the lower leg in two (3.8%) patients, and at the level of the thigh in one (1.9%) patient with wet gangrene.

The frequency of amputations directly depended on the nature of the limb lesion, the degree of ischaemia, and the depth of the foot lesion according to the Wagner classification (*p* = 0.020). Amputation was performed in 53.3% (8 of 15) of patients who had a Wagner grade IV at first hospitalization, 32.1% (9 of 28) of those with Wagner grade III lesions, and no amputations were performed with patients with Wagner grade II lesions.

During the year, death from cardiovascular events was registered in six patients (11.3%); five patients died during the year from acute myocardial infarction and/or increasing heart failure, and one patient died from a cerebral ischaemic stroke that developed in the system of the left middle cerebral artery. In 2 out of 6 patients who died during the year, limb amputation was performed due to wet gangrene. Survivors (discharged from the hospital) within a year with acute cardiovascular events included two patients with stroke (one of whom underwent an amputation) and three patients who were discharged.

Nonhealing of a diabetic foot ulcer (deterioration of the course of the wound or the absence of positive dynamics during the year despite the ongoing treatment methods) was recorded in seven patients (13.2%). In all cases, treatment was continued without amputation and limited to autodermoplasty with a perforated skin flap.

Thus, an unfavourable outcome was established in 28 patients (52.8%), and a favourable outcome was established in 25 (47.2%).

### 3.3. Histological and Immunohistochemical Examination of Wound Tissues

The analysis of histological and immunohistochemical parameters determined on the first day of hospitalization revealed many significant differences between patients with favourable and unfavourable outcomes. These differences concerned both tissues and cellular structures in all sections of the wounds we studied (edges and bottom of wounds).

#### 3.3.1. Histological Assessment of Wound Tissues Obtained during Primary Surgical Processing of Wounds in Patients with Different Outcomes of DFS

Wound tissues on the first day of hospitalization were mainly represented by leukocyte-necrotic masses, which were found in all wounds. Leukocyte-necrotic masses (or leukocyte-necrotic detritus) were identified by the accumulation of neutrophils and dead tissues and cells. Thus, a “conglomerate” of devitalized tissues (not morphologically identifiable) and leukocytes were visualized. On average (Me), in different fields, 68–86% fell to their share. It is interesting to note that at the edges of the wounds, leukocyte infiltration was significantly 1.27 times higher with a favourable outcome of DFS treatment (average 86.2% vs. 67.7%; *p* = 0.011), while in the centre of the wounds, there were 1.30 times as many leukocyte-necrotic masses in patients with an unfavourable outcome of DFS treatment (68.1% vs. 88.5%; *p* = 0.003).

Given the long-term nature of diabetic ulcers in our patients, granulation tissue was identified two times less often than leukocyte-necrotic tissue (on average, in 7–32% of fields in different sections of the wound tissue preparations), and in 14 (26.4%) wounds, granulation tissue was completely absent. When analysing the frequency of representation of granulation tissue for the different outcomes of DFS, statistically significant differences were identified. At the edges of the wounds, granulation tissue was significantly 3.59 times more likely in patients with a favourable outcome of DFS treatment (average 26.6% vs. 7.4%; *p* = 0.007), while in the centre of the wounds, granulation tissue was significantly 3.57 times more likely in patients with an unfavourable outcome of DFS treatment (8.9% vs. 31.8%; *p* = 0.001). At the edges of the wounds, the absence of granulation was observed in eight wounds (one unfavourable and seven favourable outcomes), while at the bottom of the wounds, in contrast, among eight preparations without signs of granulation, in six patients, the outcome was unfavourable. Therefore, the predictor of an unfavourable outcome to the greatest extent is the absence of granulations (and their weak severity) at the bottom of the wounds.

**Figure 3:**
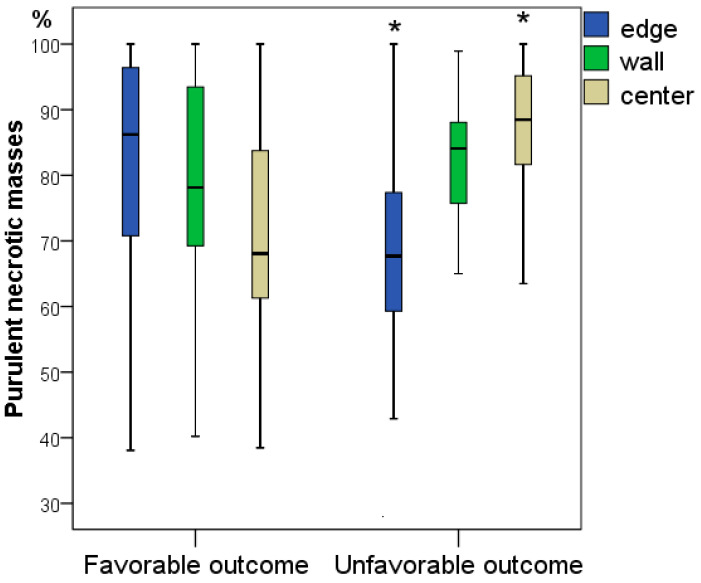
Frequency of representation of leukocyte-necrotic tissues in different parts of wounds obtained during the primary surgical treatment for patients with a favourable and unfavourable outcomes of diabetic foot treatment (*—difference between groups in terms of outcome at *p* < 0.05; Mann–Whitney U test).

**Figure 4:**
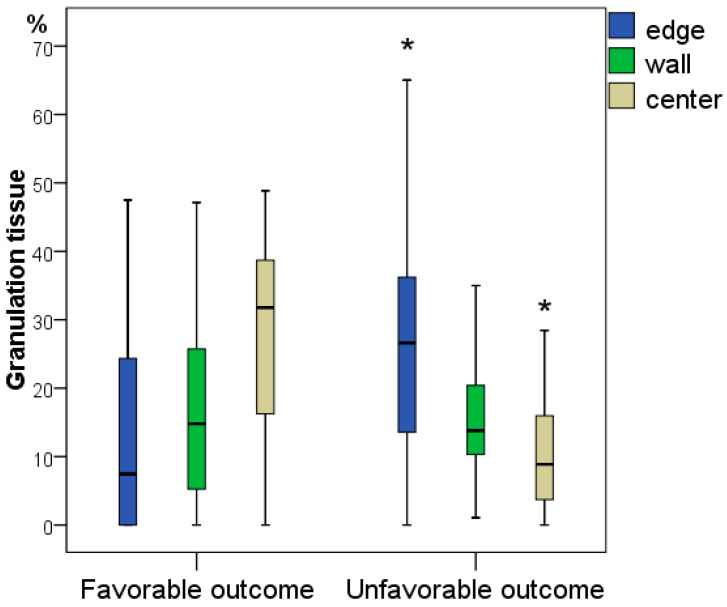
Frequency of representation of granulation tissue in different parts of wounds obtained during the primary surgical treatment for patients with a favourable and unfavourable outcomes of diabetic foot treatment (*—difference between groups in terms of outcome at *p* < 0.05; Mann–Whitney U test).

Despite the absence of significant differences in the representation of leukocyte-necrotic and granulation tissues in the wound wall, its content determines the pathophysiological dynamics, which is multidirectional for different outcomes of the diabetic foot (Figure 3 and Figure 4).

Regardless of the duration of the ongoing chronic inflammatory process, connective tissue was rarely identified. Loose connective tissue at the edge of wounds was visualized in 26 (49.1%) wounds; at the bottom, it was visualized in 14 (26.4%) preparations. On average (Me), the percentage of representation of connective tissue was 0–0.9%. In 21 wounds (39.6%), loose connective tissue was absent in all sections of the wounds, and this was not associated with the outcome (9 wounds in patients with a favourable outcome and 12 wounds in patients with an unfavourable outcome, *p* = 0.611). Dense connective tissue of the DFS wounds was even rarer (on average 0–0.7% of the preparations) and was also not associated with the outcome.

The rarest type of tissue was epithelial. Histological study of the epithelium in wound tissues (ranging from single epithelial cells to stratified keratinizing epithelium) revealed that epithelium in the wound margin was identified in only 13 patients (occupying 8-327 histological fields or from 0.7% to 32% of the analysed fields) and in the bottom of the wounds in 3 patients (15–48 cells or 1.3–4.8% of the fields). A large variation in the representation of different epithelial cells/tissues identified in less than 25% of patients was determined. There were no significant differences in the presence or absence of epithelial tissue in patients with favourable and unfavourable outcomes of DFS treatment (*p* > 0.05), so the null hypothesis of equality in the representation of the epithelium in different outcomes of DFS treatment cannot be rejected.

Correlation analysis showed the presence of significant relationships of adverse outcomes with the prevalence of purulent-necrotic masses (r = −0.355; *p* < 0.05) and granulation tissue (r = 0.375; *p* < 0.05) at the edges of the wounds and stronger relationships at the bottom of the wounds (r = 0.467; *p* < 0.05 and r = −0.416; *p* < 0.05, respectively).

Thus, nonhealing wounds are ¾ filled with leukocyte-necrotic tissues and 20% filled with granulation tissue with a low content of other tissues. Granulations and purulent-necrotic tissues are considered predictors of the outcome of DFS treatment, and their concentration varies in different parts of chronic long-term nonhealing diabetic ulcers. The presence of excess leukocyte filtration in the wound margin increases the likelihood of a favourable outcome.

#### 3.3.2. Immunohistochemical Study for Determining the Number and Frequency of Neutrophilic Leukocytes at the Edges, Wall, and Centre of Chronic Diabetic Wounds

In all regions of long-term nonhealing diabetic foot ulcers we studied, neutrophilic leukocytes were the most common, detected on average in 44–66% of the studied fields of wound preparations.

At the edge of the wounds, neutrophils were registered 1.31 times more often in patients with a favourable outcome (Me = 58.1%, IQR [49.3:68.4]) than in those with an unfavourable outcome of DFS treatment (Me = 44.1%, IQR [38.7:54.7]), and the difference was significant (*p* = 0.036; Mann–Whitney U test). In the centre of the wounds, neutrophils were registered 1.36 times more often with an unfavourable outcome (Me = 65.7%, IQR [56.4:70.8]) than with a favourable outcome (Me = 48.2%, IQR [39.1:61.8] per cent), and the difference was significant (*p* = 0.001; Mann–Whitney test). Comparative analysis of neutrophils in the intermediate section of the wound wall revealed a trend towards an increase in the number of neutrophils in patients with an unfavourable outcome by 1.15 times (Me = 66.1%, IQR [57.4:70.4] per cent) compared with that in patients with a favourable outcome (Me = 57.3%, IQR [46.5:66.7] per cent, *p* = 0.087).

**Figure 5:**
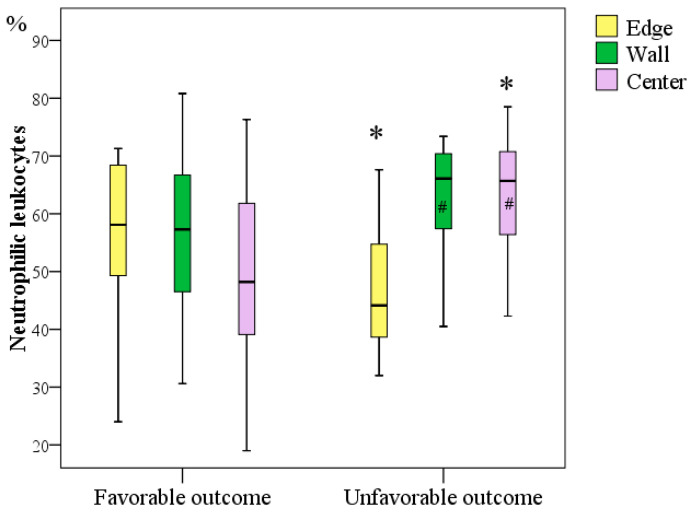
Frequency of occurrence of neutrophilic leukocytes in chronic diabetic foot wounds in patients with different outcomes on Day 1 during primary surgical treatment (*—difference between groups in terms of outcome at *p* < 0.05, Mann–Whitney U test; #—a significant increase in the number of cells relative to their content in the centre of the wounds within one outcome subgroup at *p* < 0.05, Friedman test).

A significant difference in the content of neutrophils in the bottom, walls, and edges of wounds was determined in the analysis of the entire sample of patients (*p* = 0.036; Friedman’s test). However, when separately comparing the number of neutrophils in different parts of the wounds, a significant difference was determined only in the subgroup of patients with an unfavourable outcome of DFS treatment (*p* = 0.003; Friedman’s criterion), in which, at the bottom of the wound, there were on average 1.49 times more neutrophils than at the edge of the wounds (*p* = 0.001, Bonferroni corrected) and 1.50 times more neutrophils than in the wound wall (*p* = 0.002, taking into account the Bonferroni correction). Comparison of the number of neutrophils in different sections of wounds in patients with a favourable outcome did not reveal any differences (*p* = 0.469; Friedman’s test), which indicates homogeneous inflammatory activity in all sections of the wounds in these patients (Figure 5).

Correlation analysis showed that adverse outcomes were significantly related to the concentration of neutrophilic leukocytes at the edge of wounds (r = −0.292; *p* < 0.05) and more strongly related to the concentration of neutrophilic leukocytes at the bottom (centre) of the wounds (r = 0.462; *p* < 0.05).

Thus, the frequency of neutrophilic leukocytes in DFS tissues may be a predictor of outcome. An unfavourable outcome is predetermined by the unevenness of their content with a significant increase in neutrophils at the bottom of the wounds. A uniform distribution of neutrophils with a decreasing tendency towards the centre of the wounds predetermined a favourable outcome of the treatment of DFS.

#### 3.3.3. Immunohistochemical Study for Determining the Number and Frequency of Fibroblasts at the Edges, Wall, and Centre of Chronic Diabetic Wounds

Fibroblasts were identified in all wounds. They were identified in 49 (92.5%) preparations of wound edges (visualized in 0.9–60.7% of fields), in 50 (94.3%) preparations of wound walls (determined in 0.5–47.5% fields), and in 45 (84.9%) preparations of wound centres (located at the bottom of wounds in 0.9–42% of the studied fields). Although there were no fibroblasts in four edges, three walls and eight preparations of the bottom of the wounds, there was not a single case where fibroblasts were not found in the wound among all parts of the wound.

A comparative analysis of the frequency of detection of fibroblasts with different outcomes in different parts of the wounds revealed significant differences, indicating their highest content in the centre of the wounds in patients with a favourable outcome of DFS, while at the edge, in contrast, there were more fibroblasts in patients with an unfavourable outcome of DFS.

Specifically, at the edge of wounds, fibroblasts were observed 1.77 times more often in patients with an unfavourable outcome (Me = 15.4%, IQR [38.7:54.7]) than in patients with a favourable outcome of DFS (Me = 8.7%, IQR [1.9:19.7]), and the difference was significant (*p* = 0.023; Mann–Whitney U test). At the bottom of the wounds, in contrast, fibroblasts were registered 2.38 times more often in patients with a favourable outcome (Me = 15.7%, IQR [4.6:19.7]) than in patients with an unfavourable outcome (Me = 6.6%, IQR [2.9:14.2]), and the difference was significant (*p* = 0.017; Mann–Whitney U test). Comparative analysis of fibroblasts in the wound wall did not reveal statistically significant differences, although fibroblasts were on average 1.23 times more frequently observed in patients with a favourable outcome (10.6% vs. 8.6%, *p* > 0.05).

**Figure 6:**
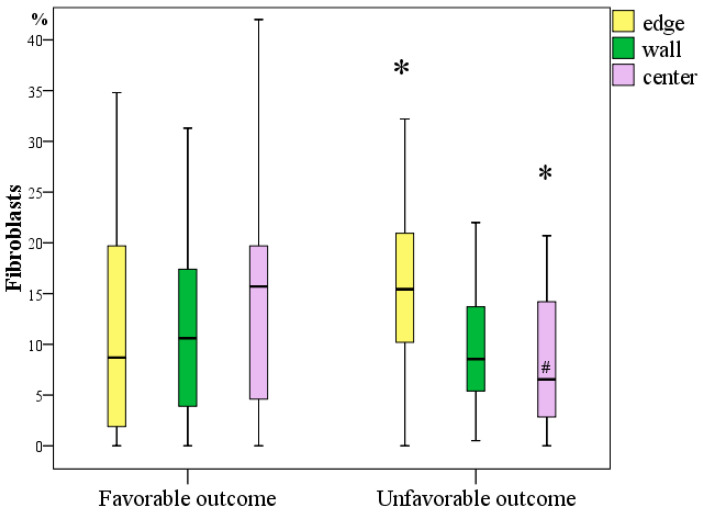
Frequency of occurrence of fibroblasts in chronic diabetic foot wounds with different outcomes on Day 1 during primary surgical treatment (*—difference between groups in terms of outcome at *p* < 0.05, Mann–Whitney U test; #—a significant increase in the number of cells relative to their content in the centre of the wounds within one outcome subgroup at *p* < 0.05, Friedman test).

When analysing the entire sample, there were no differences in the content of fibroblasts in different parts of the wounds (*p* = 0.296; Friedman’s test). However, in a separate analysis, a significant difference was determined only in the subgroup of patients with an unfavourable outcome of DFS treatment (*p* = 0.012; Friedman’s test), in which fibroblasts at the edges of the wounds were, on average, 2.33 times more frequently observed than in the centre of the wounds (*p* = 0.01, Bonferroni corrected). A comparison of the number of fibroblasts in different sections of wounds in patients with a favourable outcome did not reveal any differences (*p* = 0.613; Friedman’s test), which indicates the same regenerative activity in all sections of the wounds in these patients (Figure 6).

Correlation analysis showed a significant relationship between adverse outcomes with the concentration of fibroblasts in the wound margin (r = 0.237; *p* < 0.05) and a stronger inverse relationship with the concentration of fibroblasts in the centre of the wound (r = −0.372; *p* < 0.05).

Thus, the frequency of occurrence of fibroblasts in DFS tissues may be a predictor of outcome. An unfavourable outcome is predetermined by the unevenness of their content with a significant decrease in their number in the centre of the wounds. A uniform distribution of fibroblasts with an increasing tendency towards the centre of the wounds predetermined a favourable outcome of the treatment of DFS.

#### 3.3.4. Immunohistochemical Study to Determine the Number and Prevalence of Endothelial Cells at the Edges, Wall, and Bottom of the Diabetic Wounds

Endothelial cells were identified in virtually all wounds, specifically in 45 (85.0%) preparations of wound edges (visualized in 0.4–36.1% of fields), in 50 (94.3%) preparations of wound walls (determined in 0.2–28.6% of fields), and in 45 (84.9%) preparations of wound centres (located at the bottom of wounds in 0.5–38.9% of the studied fields). Although there were no fibroblasts in eight edges, three walls, and eight preparations of the bottom of the wounds, endotheliocytes were not found in all parts of the wounds in only one patient. In the remaining patients, endotheliocytes were present in at least one part of the wound.

A comparative analysis of the frequency of detection of endotheliocytes with different outcomes in different parts of the wounds revealed significant differences, with their highest content in the centre of the wounds in patients with a favourable outcome of DFS treatment, while at the edge, in contrast, they were more common in patients with an unfavourable outcome of DFS treatment.

Specifically, 2.96 times more endotheliocytes were registered in the wound margin in patients with an unfavourable outcome (Me = 13.9% at the interquartile interval [8.1:20.9]) than in patients with a favourable outcome of DFS treatment (Me = 4.7% at the interquartile interval [1.9:12.5]), and the difference was significant (*p* = 0.004; Mann–Whitney U test). In contrast, in the bottom of the wounds, endotheliocytes were registered 3.37 times more often in patients with a favourable outcome (Me = 17.2% at the interquartile interval [5.1:21.7]) than in patients with an unfavourable outcome (Me = 5.1% at the interquartile interval [2.2:7.0]), and the difference was significant (*p* = 0.002; Mann–Whitney U test). A comparative analysis of the prevalence of endotheliocytes in the wall of wounds did not reveal statistically significant differences; on average (median), this indicator was 8.4–8.5% regardless of the outcome of DFS treatment.

**Figure 7:**
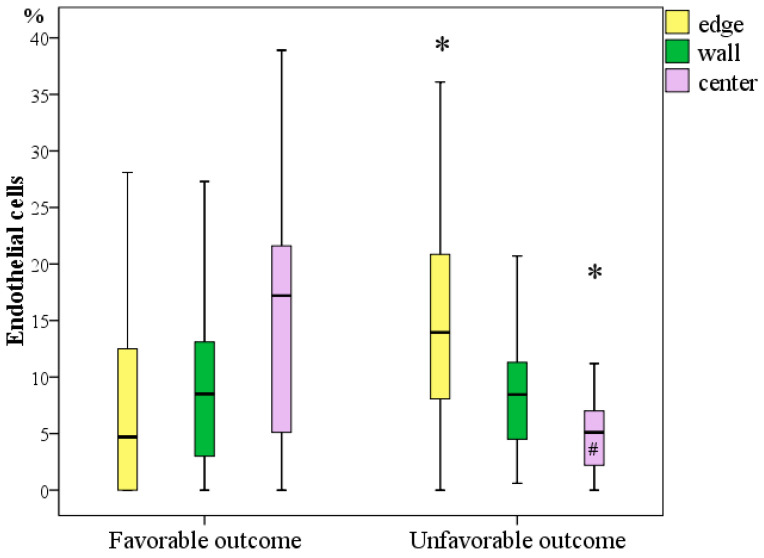
Frequency of occurrence of endotheliocytes in chronic diabetic foot wounds with different outcomes on Day 1 during primary surgical treatment (*—difference between groups in terms of outcome at *p* < 0.05, Mann–Whitney U test; #—a significant increase in the number of cells relative to their content in the centre of the wounds within one outcome subgroup at *p* < 0.05, Friedman test).

When analysing the entire sample, there were no differences in the content of endotheliocytes in different parts of the wounds (*p* = 0.942; Friedman’s test). However, a separate analysis revealed a significant difference only in the subgroup of patients with an unfavourable outcome of DFS treatment (*p* = 0.024; Friedman’s test), in which endotheliocytes at the edges of the wounds were on average 2.72 times more frequent than in the centre of the wounds (*p* = 0.023, Bonferroni corrected). A comparison of the number of endotheliocytes in different sections of wounds in patients with a favourable outcome did not reveal any differences, which indicates the same prevascularization activity in all sections of the wounds in these patients (Figure 7).

Correlation analysis showed the presence of significant relationships between adverse outcomes and the concentration of endotheliocytes in the wound margin (r = 0.402; *p* < 0.05) and a stronger inverse relationship with the concentration of endotheliocytes at the bottom of the wounds (r = −0.420; *p* < 0.05).

Thus, the frequency of endothelial cells in DFS tissues may be a third morphometric predictor of treatment outcomes. An unfavourable outcome is predetermined by the unevenness of endothelial cell content with a significant decrease in their number in the centre of the wounds. A uniform distribution of endothelial cells with an increase towards the centre of the wounds is correlated with a favourable outcome of the DFS treatment.

## 4. Discussion

Diabetic foot ulcers are complexes of anatomical and functional changes that develop against the background of late complications of diabetes (diabetic neuropathy, micro- and macroangiopathy, osteoarthropathy), contributing to increased trauma and infection of the soft tissues of the foot and the development of purulent-necrotic processes, which in advanced cases leads to necrosis of the tissues of the foot, gangrene, and amputation. Despite improved diagnostic and preventive examinations and the introduction of increasingly effective methods of treatment, the number of patients with this pathology continues to grow exponentially, taking on all the signs of an epidemic. This defines this pathology as medically and socially significant, highly disabling, and associated with global economic losses [4,6,8,10,13,14,19].

In our comprehensive study, a large percentage of had unfavourable outcomes in the treatment of DFS (53%, including 32% amputations, 13% nonhealing DFS, and 11% deaths from cardiovascular events within a year). Other studies performed at different times in different countries also found a high incidence of unfavourable outcomes in patients with DFS [8,10,14,16,19]. Such a high rate of adverse outcomes within just one year of patient follow-up highlights not only the medical but also the social significance of the problem of diabetes and, in particular, DFS.

The instrumental and clinical methods used today in clinical practice for assessing the condition of patients suffering from diabetic foot for a long time cannot always successfully predict the result of treatment [13,15,16,17,18]. Therefore, the search for additional indicators is, of course, an urgent task. To this end, as such a method the authors of that study took a histomorphological study of the tissues of a chronic ulcer obtained during the primary surgical treatment of a wound, which is standard in the management of these patients, and compared the histological results obtained with other treatment prognosis criteria used today.

The histological and morphometric picture, namely, the uniform distribution of cells of different functional types in all parts of the wounds, was fundamental in the healing of DFS and the outcome of treatment. Violation of the harmony of healing and mismatch of the regenerative processes occurring differently at the edges and bottom of the wounds did not lead to recovery but only to the preservation of inflammatory and destructive processes.

With significant imbalances of these processes at the edge and centre of the wounds, the probability of an adverse outcome in the treatment of DFS (amputation, death from cardiovascular events, and/or nonhealing of the ulcer within a year) significantly increased.

Histological examination of the wound tissues found that nonhealing wounds were ¾ filled with leukocyte-necrotic tissues and 20% filled with granulation tissue with a low content of other tissues. Granulations and purulent-necrotic tissues were predictors of the outcome of DFS, and their concentration varied in different parts of chronic long-term nonhealing diabetic ulcers. The presence of excess leukocyte infiltration in the wound margin increased the likelihood of a favourable outcome.

The immunohistochemical study revealed that the healing of chronic wounds occurs in different ways. In patients with a favourable outcome, an increase in the number of fibroblasts and endotheliocytes, as well as a decrease in the number of neutrophils, were recorded from the edge to the centre of the wounds. When the outcome was unfavourable, in contrast, an increase in neutrophils was recorded from the edge to the centre of the wound, while the frequency of fibroblasts (from 15.4% to 6.6%) and endotheliocytes (from 13.9% to 5.1%) decreased. Thus, an unfavourable outcome is predetermined by a significant, two-times or greater reduction in the frequency of fibroblasts and endotheliocytes from the edge to the centre of the wound and a low frequency of fibroblasts (<6.6%) and endothelial cells (<5.1%) in the centre of the wounds with a high content of neutrophils (>65.7%), while at the edge of the wounds, in contrast, a high content of endotheliocytes (>13.9%) and fibroblasts (>15.4%) with a relatively low frequency of neutrophilic leukocytes (<44.2%).

Thus, more attention should be paid to the biological material obtained during routine PST of a diabetic foot ulcer, since it turned out that the study of the cellular landscape of the wound adds a lot to our understanding of the timing and success of ulcer healing, and therefore can potentially change the tactics of managing a patient with a change in the pharmacological effectiveness of the means used and assistance in the timing and choice of surgical strategies. Ideally, the surgical treatment should be used earlier in cases of unfavourable outcome risk, instead of waiting for the ulcer to heal for several more months. This approach may ultimately help reduce the financial costs of treatment.

We would like to believe that our study will be a step towards a more holistic understanding of the pathogenesis of chronic wound regeneration and personalized therapy for DFS. In addition, this study will provide an opportunity to adequately and competently plan and conduct a future multicentre study on DFS with a calculation of the planned number of patients and effect size.

## 5. Conclusions

When conducting primary surgical treatment for patients with DFS, it is advisable to conduct a histological examination of the tissues obtained with a differential analysis of the cell landscape at the edge and centre of the wounds. Particular attention should be given to the prevalence of leukocytes, fibroblasts, and endotheliocytes.

An uneven distribution of cellular elements across the wound is significantly more common in patients with an unfavourable outcome of treatment, while a relatively uniform distribution in different parts of the wound is more common in patients with a favourable outcome.

### Limitations

The population for the study was homogeneous, but not very large. This work excludes the factor of multiple clinics, surgeons, treatment regimens, etc. by selecting all the patients from only one specialized centre for the treatment of diabetic foot ulcers. All the patients were observed only by the head of the centre (Dr. Konstantin Koreyba), who monitored, conducted, and performed all diagnostic and therapeutic operations. It is noteworthy that this is a centre that serves a large region of the Russian Federation i.e., the central part of the Republic of Tatarstan of the Russian Federation, along a radius of about 150 km. The study was limited to selected patients who were ready to comply with all the recommendations and avail support such as the possibility of selecting regular orthopaedic shoes and timely dressings. Additionally, expensive immunohistochemical studies significantly reduced the sample size.

## Data Availability

Not applicable.

## References

[1] McCoy M.A., Theeke L.A. (2019). A systematic review of the relationships among psychosocial factors and coping in adults with type 2 diabetes mellitus. Int. J. Nurs. Sci..

[2] Singh S., Bansal A., Singh V., Chopra T., Poddar J. (2022). Flavonoids, alkaloids and terpenoids: a new hope for the treatment of diabetes mellitus. J. Diabetes Metab. Disord..

[3] Lee W.J., Almalki O. (2017). Recent advancements in bariatric/metabolic surgery. Ann. Gastroenterol. Surg..

[4] Dahlén A.D., Dashi G., Maslov I., Attwood M.M., Jonsson J., Trukhan V., Schiöth H.B. (2022). Trends in Antidiabetic Drug Discovery: FDA Approved Drugs, New Drugs in Clinical Trials and Global Sales. Front. Pharmacol..

[5] Horton W.B., Barrett E.J. (2021). Microvascular Dysfunction in Diabetes Mellitus and Cardiometabolic Disease. Endocr. Rev..

[6] Zheng Y., Ley S.H., Hu F.B. (2018). Global aetiology and epidemiology of type 2 diabetes mellitus and its complications. Nat. Rev. Endocrinol..

[7] Antonetti D.A., Silva P.S., Stitt A.W. (2021). Current understanding of the molecular and cellular pathology of diabetic retinopathy. Nat. Rev. Endocrinol..

[8] Viigimaa M., Sachinidis A., Toumpourleka M., Koutsampasopoulos K., Alliksoo S., Titma T. (2020). Macrovascular Complications of Type 2 Diabetes Mellitus. Curr. Vasc. Pharmacol..

[9] Richardson A., Park W.G. (2021). Acute pancreatitis and diabetes mellitus: a review. Korean J. Intern Med..

[10] Lavery L.A., Oz O.K., Bhavan K., Wukich D.K. (2019). Diabetic Foot Syndrome in the Twenty-First Century. Clin. Podiatr. Med. Surg..

[11] Riedel U., Schüßler E., Härtel D., Keiler A., Nestoris S., Stege H. (2020). Wundbehandlung bei Diabetes und diabetischem Fußulkus [Wound treatment in diabetes patients and diabetic foot ulcers]. Hautarzt.

[12] Navarro-Flores E., Cauli O. (2020). Quality of Life in Individuals with Diabetic Foot Syndrome. Endocr. Metab. Immune Disord. Drug. Targets.

[13] Rossboth S., Lechleitner M., Oberaigner W. (2020). Risk factors for diabetic foot complications in type 2 diabetes—A systematic review. Endocrinol. Diabetes Metab..

[14] Brennan M., Sutherland B., Musuuza J., Smith B., Balasubramanian P., Kurter S., Crnich C., Safdar N. (2018). 2372. Multidisciplinary Care Teams to Reduce Major Amputations for Patients With Diabetic Foot Ulcers: A Systematic Review. Open Forum. Infect. Dis..

[15] Ferreira L., Carvalho A., Carvalho R. (2018). Short-term predictors of amputation in patients with diabetic foot ulcers. Diabetes Metab. Syndr..

[16] Jeon B.J., Choi H.J., Kang J.S., Tak M.S., Park E.S. (2017). Comparison of five systems of classification of diabetic foot ulcers and predictive factors for amputation. Int. Wound J..

[17] Gülcü A., Etli M., Karahan O., Aslan A. (2020). Analysis of routine blood markers for predicting amputation/re-amputation risk in diabetic foot. Int. Wound J..

[18] Jeong E.G., Cho S.S., Lee S.H., Lee K.M., Woo S.K., Kang Y., Yun J.S., Cha S.A., Kim Y.J., Ahn Y.B. (2018). Depth and combined infection is important predictor of lower extremity amputations in hospitalized diabetic foot ulcer patients. Korean J. Intern Med..

[19] Stupin V.A., Silina E.V., Koreyba K.A., Goryunov S.V. (2019). Diabetic foot Syndrome (Epidemiology, Pathophysiology, Diagnosis and Treatment).

[20] Ashrafzadeh S., Hamdy O. (2019). Patient-Driven Diabetes Care of the Future in the Technology Era. Cell Metab..

[21] Chung W.K., Erion K., Florez J.C., Hattersley A.T., Hivert M.F., Lee C.G., McCarthy M.I., Nolan J.J., Norris J.M., Pearson E.R. (2020). Precision Medicine in Diabetes: A Consensus Report From the American Diabetes Association (ADA) and the European Association for the Study of Diabetes (EASD). Diabetes Care.

[22] Landgraf R., Aberle J., Birkenfeld A.L., Gallwitz B., Kellerer M., Klein H., Müller-Wieland D., Nauck M.A., Reuter H.M., Siegel E. (2019). Therapy of Type 2 Diabetes. Exp. Clin. Endocrinol. Diabetes.

[23] Koreyba K.A., Tsyplakov D.E., Kadyrov R.K., Stupin V.A., Silina E.V., Feiskhanov A.K., Blatt N.L., Nedosugov A.A. (2022). Medical imaging in surgery for diabetic foot syndrome: Clinical and morphological case report. BioNanoScience.

[24] Avtandilov G.G. (1990). Meditsinskaya morfometriya (Medical Morphometry).

[25] Tsyplakov D.E., Shakirova A.Z., Silina E.V. (2023). Macro- and microscopic diagnosis in practical classes in pathological anatomy. Atlas: Tutorial Guide.

[26] Świątoniowska N, Sarzyńska K, Szymańska-Chabowska A, Jankowska-Polańska B (2019). The role of education in type 2 diabetes treatment. Diabetes Res. Clin. Pract..

[27] Chatterjee S., Davies M.J., Heller S., Speight J., Snoek F.J., Khunti K. (2018). Diabetes structured self-management education programmes: a narrative review and current innovations. Lancet Diabetes Endocrinol..

[28] Nassar C.M., Montero A., Magee M.F. (2019). Inpatient Diabetes Education in the Real World: an Overview of Guidelines and Delivery Models. Curr. Diabetes Rep..

[29] Greenwood D.A., Gee P.M., Fatkin K.J., Peeples M. (2017). A Systematic Review of Reviews Evaluating Technology-Enabled Diabetes Self-Management Education and Support. J. Diabetes Sci. Technol..

[30] Rajpal A., Ismail-Beigi F. (2020). Intermittent fasting and ’metabolic switch’: Effects on metabolic syndrome, prediabetes and type 2 diabetes. Diabetes Obes. Metab..

[31] Clifton P. (2019). Metabolic Syndrome-Role of Dietary Fat Type and Quantity. Nutrients.

[32] Nolan C.J., Prentki M. (2019). Insulin resistance and insulin hypersecretion in the metabolic syndrome and type 2 diabetes: Time for a conceptual framework shift. Diabetes Vasc. Dis. Res..

